# Timing of initiation of renal replacement therapy in acute kidney injury: an updated meta-analysis of randomized controlled trials

**DOI:** 10.1080/0886022X.2019.1705337

**Published:** 2020-01-02

**Authors:** Ling Zhang, Dezheng Chen, Xin Tang, Peiyun Li, Yong Zhang, Ye Tao

**Affiliations:** a Department of Nephrology, West China Hospital of Sichuan University, Chengdu, China; b Department of Nephrology, Jianyang People’s Hospital of Sichuan Provinces, Jianyang, China

**Keywords:** Acute kidney injury, renal replacement therapy, timing, early strategy, meta-analysis

## Abstract

**Purpose:**

The results from randomized controlled trials (RCTs) concerning the timing of initiation of renal replacement therapy (RRT) for patients with acute kidney injury (AKI) are still inconsistent.

**Materials and methods:**

We searched for RCTs, as well as relevant references, focusing on the timing of RRT for AKI patients in the Medline, Embase, Cochrane Library, Google Scholar and Chinese databases from their inception to December 2018.

**Results:**

We included 18 RCTs from 1997 to 2018 involving 2856 patients. Pooled analyses of all RCTs showed no significant difference in mortality between early initiation and delayed initiation of RRT (RR 0.98, 95% CI: 0.89 to 1.08, *p* = .7) (*I*^2^ = 2%), and similar results were found in critically ill and community-acquired AKI patients, as well as in a subgroup of patients with sepsis and in cardiac surgery recipients. There was also no difference in the incidence of dialysis independence (RR 0.75, 95% CI: 0.47 to 1.2, *p* = .2) (*I*^2^ = 0). However, an early RRT strategy was associated with a significantly higher incidence of the need for RRT for AKI patients (RR 1.24, 95% CI: 1.13 to 1.36, *p* < .01) (*I*^2^ = 34%).

**Conclusions:**

As no life-threatening complications occurred, there was no evidence to show any benefit of an early RRT strategy for critically ill or community-acquired AKI patients; in contrast, a delayed strategy might avert the need for RRT.

## Introduction

Because there are no effective drugs for critically ill patients with acute kidney injury (AKI), renal replacement therapy (RRT) has been the main treatment [1,2]; however, the optimal timing of initiating RRT is a key question that remains unanswered. There is no doubt that RRT should be performed when AKI patients are complicated with life-threatening hyperkalaemia, metabolic acidosis or acute pulmonary edema. However, the timing of RRT initiation for AKI patients without such complications has not yet been defined.

A previous randomized controlled trial [3] (RCT) in 2004 reported that early initiation of RRT significantly reduced the mortality of AKI patients following coronary bypass surgery. Subsequently, many observational studies [4,5] also showed that an early RRT strategy could reduce the mortality of AKI patients. A meta-analysis [6] including 2 RCTs and 13 observational studies reported that an early strategy could present a survival benefit. However, since 2013, additional RCTs [7–9] have not supported the conclusion of the previous meta-analysis, and a recent meta-analysis [10] found no survival advantage for the early initiation of RRT among high-quality RCTs and observational studies. Furthermore, the results from the latest high-quality RCTs [11–13] are still controversial.

Given the inconsistency of the existing literature and the insufficient statistical power of previous systematic reviews, which either included many observational studies or did not include the latest trials, we conducted a meta-analysis of RCTs to summarize the available evidence on the timing of initiating RRT in patients with AKI.

## Methods

We performed this systematic review using the guidelines proposed by the Cochrane Collaboration in the Cochrane Handbook for Systematic Reviews of Interventions (http://www.cochrane-handbook.org). The protocol has been registered on PROSPERO (CRD 42016042398) [14].

### Study selection criteria

#### Participants

This review focused on patients with AKI who received any modality of RRT.

#### Interventions

Because there is no consensus-driven definition of ‘‘early’’ versus ‘‘late’’ initiation of RRT, in this review, we accepted a broad definition of ‘early’ used by the original investigators in their respective studies, such as biochemical markers according to the KDIGO or RIFLE classifications or time-based cutoffs such as a defined time from ICU admission or development of a biochemical ‘start time’. A limitation of this approach is that ‘early’ according to one study investigator might be considered ‘delayed’ by another study investigator. ‘Delayed’ RRT criteria involved either conventional practice or expectant care (i.e., no RRT initiation). ‘Conventional practice’ generally involved implementing RRT following the development of classic RRT indications. There was no restriction on RRT modalities in this review, and all possible RRT modalities, including continuous replacement therapy (CRRT), intermittent hemodialysis (IHD) or peritoneal dialysis (PD), were included.

#### Types of outcome measures

The primary outcome was mortality. Dialysis independence, incidence of need for RRT and RRT complications were also analyzed.

#### Types of studies

We included all RCTs concerning the timing of initiation of RRT for patients with AKI. Non-randomized studies and studies published in abstracts, reviews, commentaries, and editorials were excluded.

### Search methods for the identification of studies

#### Study selection

We used the Cochrane risk-of-bias tool [15] to undertake, and the PRISMA (Preferred Reporting Items for Systematic Reviews and Meta-Analyses) statement methodology [16] (see Supplementary material 1) to report, a systematic review and meta-analysis of RCTs. Two independent reviewers (L.Z. and D.C.) conducted a search in Medline, Embase, the Cochrane Library, Google Scholar, a Chinese database (CNKI) and relevant journals. Trials were considered without language or date restrictions. We performed the last updated search on December 31, 2018. The following text words and corresponding heading terms were used as search terms: ‘acute kidney injury or acute renal failure’ and ‘dialysis or renal replacement therapy or CRRT or IHD or hemodialysis or hemofiltration or hemodiafiltration’ and ‘time or timing or early or earlier or accelerate or accelerated or acceleration’ and ‘random or randomly or randomized or randomization’ (see Supplementary material 2). Related articles and reference lists were manually searched to avoid omissions. After screening the titles, we evaluated the abstracts for relevance and identified them as included, excluded or requiring further assessment. At this stage, if a paper required further assessment, we contacted the study’s lead investigator by e-mail and/or telephone with a request for further information.

#### Data extraction

The inclusion criteria were as follows: (a) the study was a prospective RCT concerning the effect of timing of initiation of RRT for patients with AKI; (b) the intervention was any form of RRT as long as the only difference in the 2 arms was the timing of initiation of RRT; and (c) sufficient data were available to calculate a relative risk (RR) or standardized mean difference (SMD) with 95% confidence interval (95% CI). The following exclusion criteria were used: (a) no relevant data; (b) nonrandomized design; and (c) study was not conducted in humans. For studies with the same or overlapping data by the same authors, the most suitable study with the largest number of cases or latest publication date was selected.

Two investigators (L.Z. and D.C.) assessed each study independently and recorded the eligibility, quality and outcomes of each study. Disagreements regarding eligibility arose with 4% of the articles (*κ* = 0.91), which were resolved by a third party through consensus. A third investigator (Y.T.) provided arbitration in case of disagreement. We extracted the following information: first author, publication year, country, study design, funding, number of participants, patient population, sequential organ failure assessment (SOFA) score, fluid balance, inclusion criteria, RRT modality, RRT timing, mortality, incidence of kidney recovery, need for RRT and complications (see Tables 1 and 2 and Supplementary material 3).

**Table 1.** Characters of included studies.

Studies	Country	Center	Adult or pediatric	Patients No.	Male	Age (y)	Funding
Barbar 2018 [11]	France	Multiple	Adult	488	61%	69	Public
Bouman 2002 [17]	Netherlands	Multiple	Adult	106	60%	68	Unclear
Combes 2015 [6]	France	Multiple	Adult	224	79%	60	Mixed
Durmaz 2003 [18]	Turkey	Single	Adult	44	85%	56	Unclear
Gaudry 2016 [10]	France	Multiple	Adult	619	NR	66	Public
Jamale 2013 [7]	India	Single	Adult	208	68%	42	Public
Lu 2012 [19]	China	Single	Adult	121	60%	58	Public
Lumletgul 2018 [20]	Thailand	Multiple	Adult	118	49%	67	Public
Meersch 2017 [21]^a^	Germany	Single	Adult	230	63%	67	company
Payen 2009 [22]	France	Multiple	Adult	76	71%	58	Mixed
Pursnani 1997 [23]	India	Single	Adult	35	NR	NR	Unclear
Srisawat 2017 [24]	Thailand	Single	Adult	40	55%	67	Public
Sugahara 2004 [1]	Japan	Single	Adult	28	64%	65	Unclear
Tang 2016 [25]	China	Single	Adult	46	46%	54	Unclear
Wald 2015 [5]	Canada	Multiple	Adult	100	72%	63	Mixed
Xiao 2016 [26]	China	Single	Adult	79	66%	51	Unclear
Yin 2018 [27]	China	Single	Adult	63	67%	61	Unclear
Zarbock 2016 [9]	Germany	Single	Adult	231	63%	67	company

Mixed: mixed with public and company funding.

^a^ Study of *Meersch 2017* was the long-term (12 months) follow-up of the *Zarbock 2016* study, and we only extracted the long-term mortality data from *Meersch 2017* for meta-analysis.

**Table 2.** Patients’ population, inclusion criteria, RRT modality and RRT timing.

Studies	Patients population	Inclusion criteria	RRT modality	RRT timing in Early group	RRT timing in Delayed Group
Barbar 2018 [11]	Sepsis	AKI RIFLE-failure stage	RRT	<12 h	>48 h
Bouman 2002 [17]	Surgery	AKI under ventilation; U*O* < 30 mL/h for 6 h and Cc*r* < 20 mL/min	CRRT	<12 h	Ure*a* > 40 mmol/L, *K* > 6.5 mmol/L or severe pulmonary edema
Combes 2015 [6]	Cardiac surgery	Shock; requiring high-dose vasopressors or needing ECMO	CRRT	<24 h after randomization	AKI-AKIN stage 3, ure*a* > 36 mmol/L or life-threatening hyperkalemia
Durmaz 2003 [18]	Cardiac surgery	Sc*r* > 220 umol/L	IHD	Scr ris*e* > 10% within 48 h	Scr ris*e* > 50% or U*O* < 400ml/d
Gaudry 2016 [10]	Multisystem	AKI under ventilation; KDIGO-stage 3; requiring vasopressors	RRT	<6 h after randomization	One of the laboratory abnormalities developed or oliguria or anuria lasted for more than 72 h
Jamale 2013 [7]	Multisystem	AKI	IHD	C*r* > 619 µmol/L	Treatment-refractory hyperkalemia, volume overload, and acidosis.
Lu 2012 [19]	Multisystem	AKI with SIRS	CRRT	RIFLE-stage 1,2	RIFLE-stage 3
Lumletgul 2018 [20]	Multisystem	AKI	CRRT	<6 h after Furosemide stress test (FST)	Conventional indication for RRT
Meersch 2017 [21]	Surgery	Severe AKI	CRRT	<8 h of diagnosis of KDIGO-AKI stage 2	<12 h of diagnosis of KDIGO-AKI stage 3
Payen 2009 [22]	Sepsis	SAPS II >35	CRRT	<24 h after randomization	Conventional indication for RRT
Pursnani 1997 [23]	Multisystem	Acute tubular necrosis	IHD	Sc*r* > 619 µmol/L	Conservative therapy
Sugahara 2004 [1]	Cardiac surgery	U*O* < 30 mL/h or Scr ris*e* > 0.5 mg/dL	CRRT	U*O* < 30 mL/h	U*O* < 20 mL/h
Tang 2016 [25]	Sepsis	AKIN-AKI stage 2 or 3	CRRT	<48 h after randomization	>48 h after randomization
Wald 2015 [5]	Multisystem	Severe AKI	RRT	<24 h after randomization	*K* > 6.0 mmol/l, serum bicarbonat*e* < 10 mmol/l, or PaO_2_/FiO_2_ <200 with pulmonary edema
Xiao 2016 [26]	Wasp venom poisoning	MODS	IHD	SOFA >5	AKI-RIFLE stage 2
Yin 2018 [27]	Multisystem	AKI RIFLE-failure stage	CRRT	<12 h	>48 h
Zarbock 2016 [9]	Surgery	Severe AKI	CRRT	<8 h of diagnosis of KDIGO-AKI stage 2	<12 h of diagnosis of KDIGO-AKI stage 3

AKI: acute kidney injury; Ccr: creatinine clearance rate; CRRT: continuous renal replacement therapy; ECMO: extracorporeal membrane oxygenation; FST: furosemide stress test; IHD: intermittent hemodialysis; MODS: multiple Organ Dysfunction Syndrome; RRT: renal replacement therapy; SAPS: simplified acute physiology scoring; Scr: serum creatinine; SIRS: Systemic Inflammatory Response Syndrome; SOFA: sepsis-related Organ Failure Assessment; UO: urine output.

#### Quantitative data synthesis

Independently and in duplicate, reviewers assessed the risk of bias using the Cochrane collaboration tool [15]. For each included study, a description, a comment, and a judgement of ‘high’, ‘unclear’, or ‘low’ risk of bias was provided for each of the following domains: adequate random sequence generation; allocation sequence concealment; blinding of participants and personnel; blinding for objective outcomes; incomplete outcome data; free of selective outcome reporting; and free of other bias. Studies with a high risk of bias for any one or more key domains were considered to have high risk of bias. Studies with a low risk of bias for all key domains were considered to have low risk of bias. Otherwise, they were considered to have unclear risk of bias. Studies with low risk of bias were considered high-quality studies.

Before the analysis, data were standardized into equivalent units. For dichotomous variables, mortality in the experimental and control groups was expressed as RR and 95% CI. For continuous variables, SMD and 95% CI were calculated for each study. Heterogeneity was evaluated using the Cochrane Q test and the *I*^2^ statistic to assess the degree of inter-study variation. *I*^2^ values of 0 to 24.9%, 25 to 49.9%, 50 to 74.9%, and 75 to 100% were considered as having no, mild, moderate, and significant thresholds for statistical heterogeneity, respectively [23,28]. A random-effects model was performed to provide more conservative estimates of effect in the presence of known or unknown heterogeneity. Subgroup analyses were carried out for different follow-up, different populations, different RRT modalities and study qualities. We performed a sensitivity analysis by separately pooling the most optimistic and pessimistic results from each included study. Publication bias was analyzed once sufficient RCTs (*n* > 10) were identified by visual inspection of asymmetry in Begg’s funnel plots as well as the Egger’s test [17]. Data analysis was performed using Review Manager 5.2 (RevMan, The Cochrane Collaboration, Oxford, United Kingdom) and STATA 12.0 (StataCorp, College Station, TX).

## Results

### Eligible studies

**Figure 1.**
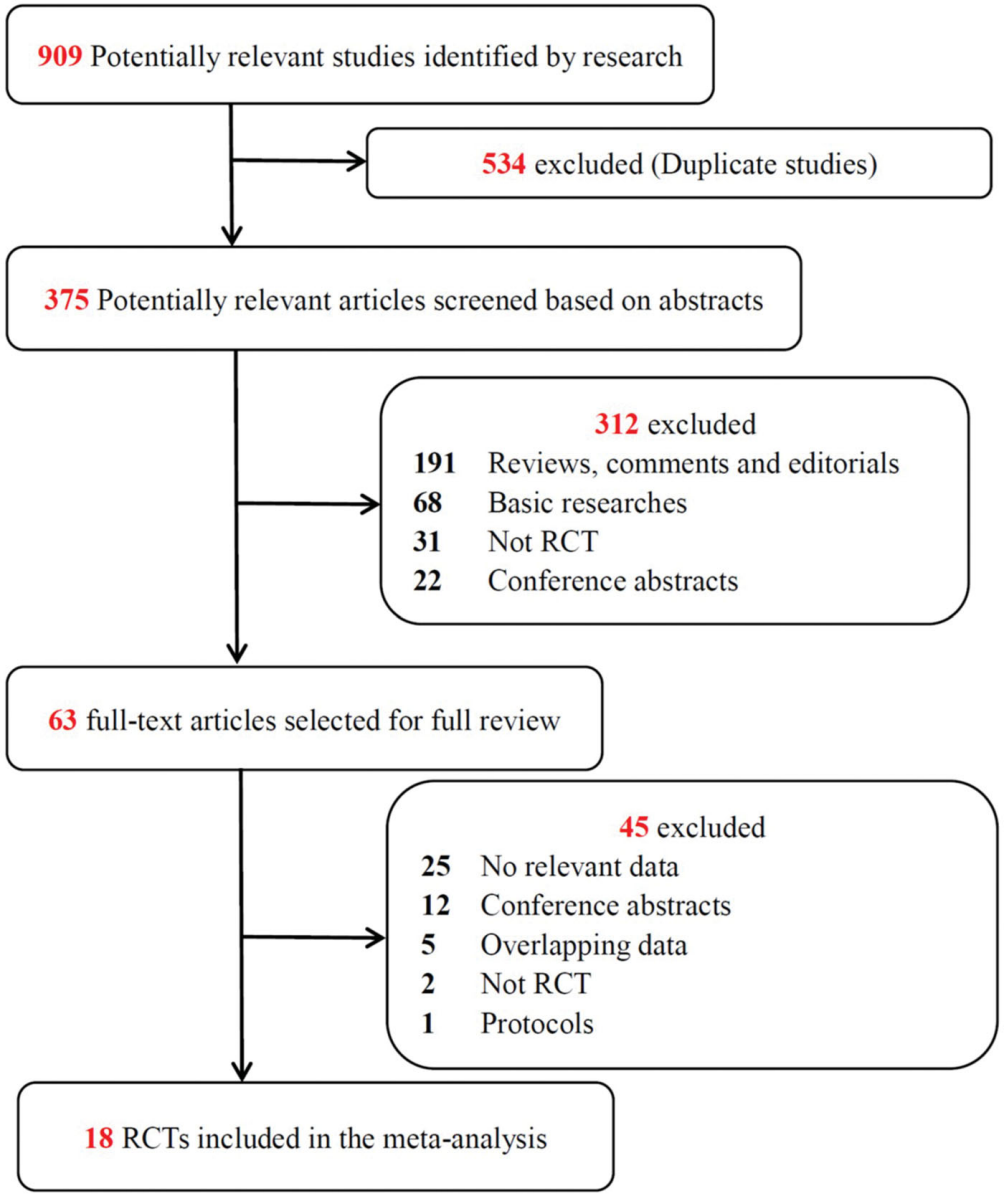
Flow chart of selection of studies.

The study selection process is presented in Figure 1. The literature search yielded 909 potentially relevant records. By screening the titles, we removed 534 duplicate studies. After the abstract of each study was evaluated, 312 studies were excluded as they did not meet the inclusion criteria. Subsequently, we carefully read the full text of each of the remaining 63 studies and excluded 45 studies for the following reasons: no relevant data (*n* = 25), conference abstracts (*n* = 12), overlapping data (*n* = 5), not an RCT (*n* = 2) and protocols (*n* = 1). Finally, 18 RCTs were included in the meta-analysis.

As shown in Table 1, the eligible studies were conducted from 1997 through 2018 with a total of 2856 patients, and the sample sizes ranged from 28 to 619 patients. All 18 studies focused on adult AKI patients. Among them, 9 studies were from Asia, 8 were from Europe, and 1 was from North America. A variety of outcomes were recorded in these studies, including mortality (*n* = 18; 100%) [3,7–9,11–13,18–22,24–27,29,30], incidence of dialysis independence (*n* = 9; 50%) [7–9,11–13,20,30], incidence of the need for RRT (*n* = 11; 61%) [7–9,11–13,19,20,22,27,30] and complications (*n* = 9; 50%) [7–9,11–13,18,22,30]. The details of the patient population, inclusion criteria, RRT modality and RRT timing are presented in Table 2.

### Assessment of methodological quality

**Figure 2.**
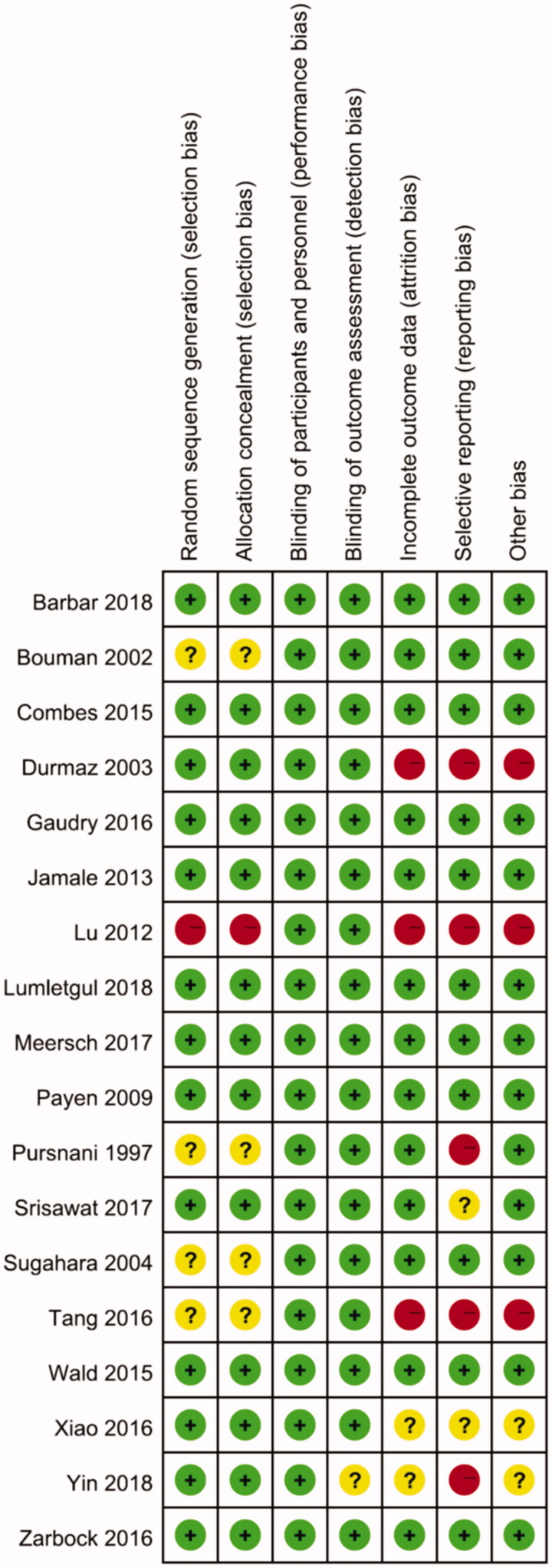
Summary of risk of bias.

The details of the risk of bias are summarized in Figure 2. Nine (50%) studies were judged to have low risk of bias, 4 to have high risk of bias, and 6 were judged to have an unclear risk of bias. Thirteen (72%) trials generated an adequate randomized sequence and reported appropriate allocation concealment. In addition, twelve (67%) studies reported funding support in the text, with seven (58%) of these supported by public funding and the other five supported by corporate or both public and corporate funding (shown in Table 1). Two studies [11,29] that were supported by corporate funding declared that the sponsors had no role in the design of the study and interpretation of the data. Among all the included studies, none were double-blinded. However, blinding of patients and clinicians is extremely difficult in studies evaluating complex interventions such as the RRT protocol, and the authors determined that the primary outcome (mortality) was not likely to be influenced by a lack of blinding.

### Mortality

**Figure 3.**
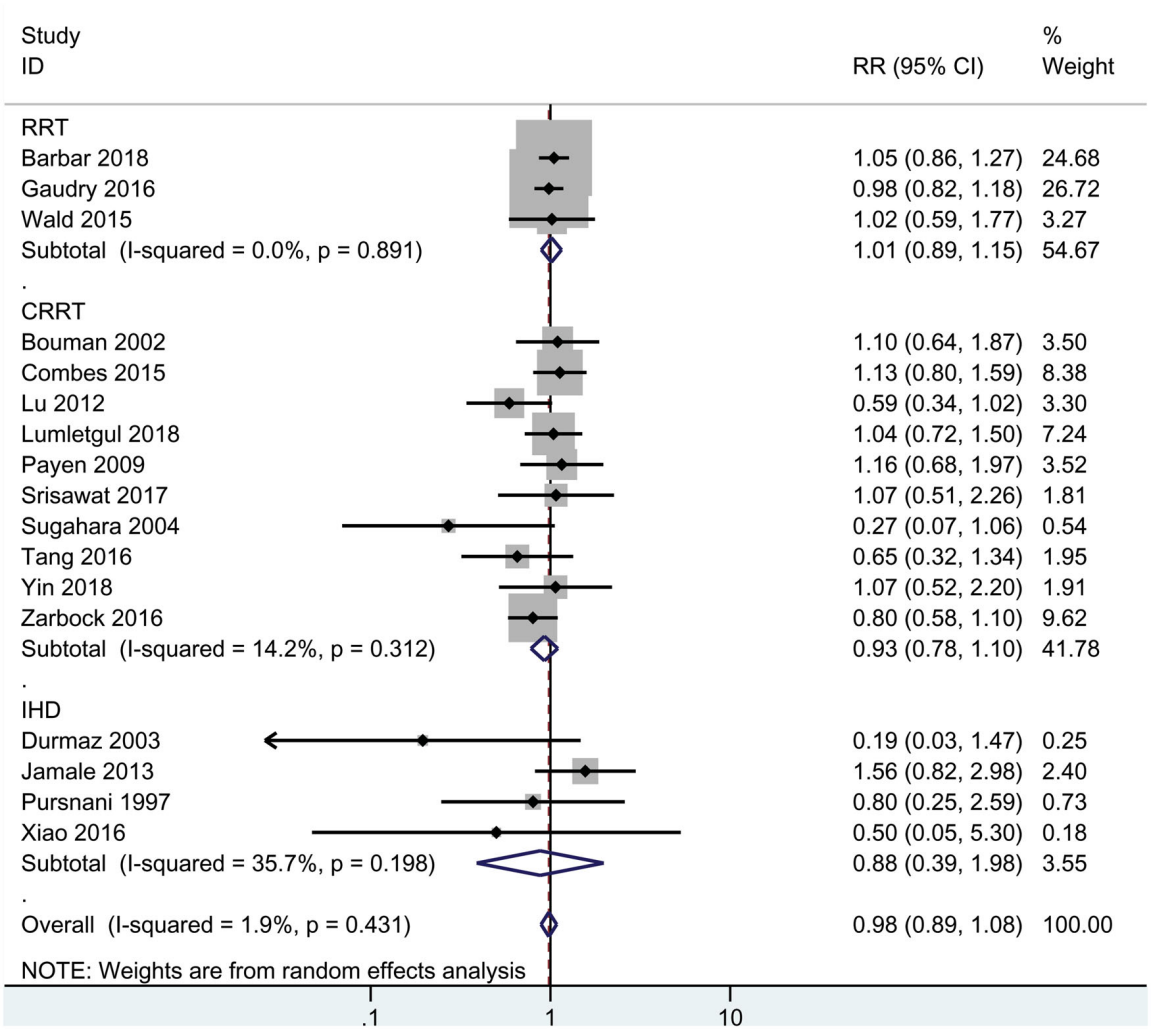
Mortality.

Overall, eighteen RCTs including 2856 patients reported data on mortality. As shown in Figure 3, the results of the summary analysis showed no significant difference in mortality between early and delayed initiation of RRT (RR 0.98, 95% CI: 0.89 to 1.08, *p* = .9), and there was no evidence of heterogeneity (*I*^2^ = 2%). Subgroup analysis is presented in Table 3. In the subgroup of high-quality RCTs [3,7,8,11,13,18,26,30], there was no difference in mortality between early and delayed RRT (RR 1.02, 95% CI: 0.92 to 1.13, *p* = .7) (*I*^2^ = 17%). There was also no significant difference in 28-day mortality (RR 0.99, 95% CI: 0.88 to 1.11), 90-day mortality (RR 1.06, 95% CI: 0.91 to 1.24) and long-term (>6-month) mortality (RR 0.94, 95% CI: 0.74 to 1.19) between early and delayed initiation of RRT.

**Table 3.** Subgroup analysis of mortality.

	No. of studies	RR (95% CI)	*I* ^2^
Overall	17	0.98 (0.89, 1.08)	2%
High-quality studies	8	1.02 (0.92, 1.13)	0
28-day mortality	9	0.99 (0.88, 1.11)	0
90-day mortality	4	1.06 (0.91, 1.24)	0
>6-month mortality	2	0.94 (0.74, 1.19)	52%
AKI with critical illness	14	0.96 (0.87, 1.06)	7%
Community-acquired AKI	3	1.27 (0.74, 2.18)	0
Patients with sepsis	3	1.03 (0.86, 1.23)	0
Patients with surgery	5	0.87 (0.61, 1.24)	50%
Patients with cardiac surgery	3	0.5 (0.15, 1.72)	69%
Modality of CRRT	10	0.93 (0.78, 1.1)	14%
Modality of IHD	4	0.88 (0.39, 1.98)	36%
SOFA score >12	6	0.97 (0.83, 1.12)	18%
SOFA score <12	5	1.02 (0.87, 1.2)	0
Positive fluid balance	6	1 (0.88, 1.15)	0
Patients in Asia	9	0.89 (0.68, 1.16)	21%
Patients in Europe	7	1 (0.89, 1.12)	0

Furthermore, similar mortality rates for early and delayed RRT were found in critically ill patients with AKI (RR 0.96, 95% CI: 0.87 to 1.06) and community-acquired AKI patients (RR 1.27, 95% CI: 0.74 to 2.18), as well as in the subgroup of patients with sepsis (RR 1.03, 95% CI: 0.86 to 1.23) and cardiac surgery recipients (RR 0.5, 95% CI: 0.15 to 1.72). Among the included studies, ten studies [3,8,11,20–22,24–26,30] focused on CRRT, 4 studies [9,18,19,27] focused on IHD, and the other 3 studies [7,12,13] used CRRT or IHD as the RRT modalities. A similar mortality rate (RR 0.93, 95% CI: 0.78 to 1.1) was found in the CRRT subgroup and in the IHD subgroup (RR 0.88, 95% CI: 0.39 to 1.98). PD was not used in the included studies.

### Dialysis independence

**Figure 4.**
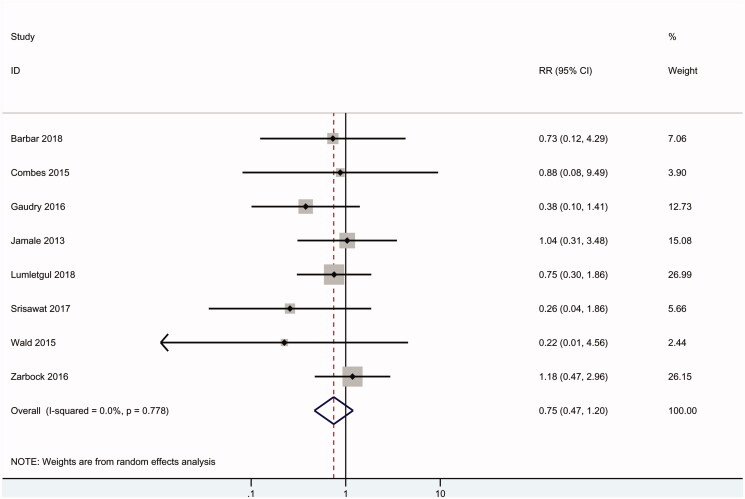
Dialysis independence of all patients.

As shown in Figure 4, nine RCTs including 1790 patients reported data on dialysis independence, and no difference was found between the early and delayed groups (RR 0.75, 95% CI: 0.47 to 1.20, *p* = .2) with no heterogeneity (*I*^2^ = 0).

### Need for RRT

**Figure 5.**
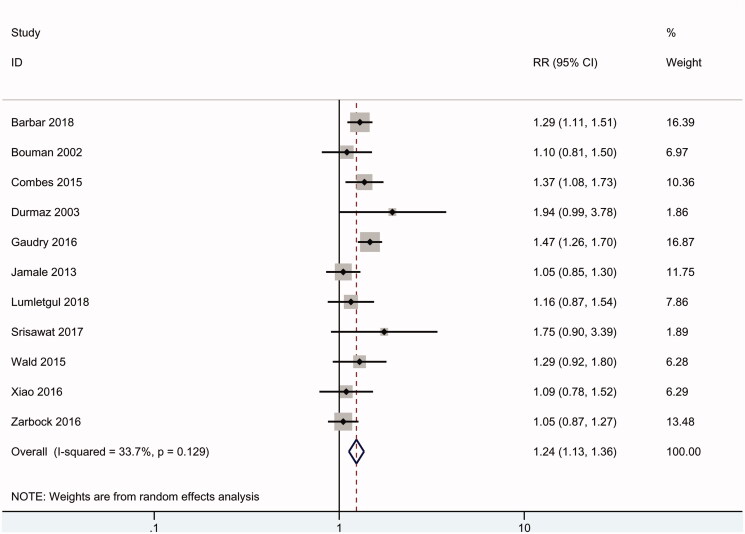
Need for RRT.

Eleven of the RCTs (2257 patients) reported data on the need for RRT. Overall, 98% of patients in the early group received RRT; in contrast, 65% of patients in the delayed group received RRT. According to the meta-analysis, an early RRT strategy was associated with a significantly higher incidence of the need for RRT in AKI patients (RR 1.24, 95% CI: 1.13 to 1.36, *p* < .01), with mild heterogeneity (*I*^2^ = 34%) (Figure 5).

### Complications

As shown in Table 4, six RCTs reported data on bleeding and showed no difference between early and delayed initiation of RRT (RR 0.89, 95% CI: 0.67 to 1.17) (*I*^2^ = 0), hypotension (RR 1.07, 95% CI: 0.9 to 1.28), hypothermia (RR 1.47, 95% CI: 0.35 to 6.27), hypophosphatemia (RR 2.34, 95% CI: 0.62 to 8.82), thrombocytopenia (RR 1.12, 95% CI: 0.87 to 1.44) and hyperkalaemia (RR 0.31, 95% CI: 0.02 to 5.69). However, four studies [7,8,12,30] reported the incidence of catheter-related complications and found that early initiation of RRT was associated with a trend of a high incidence of catheter-related complications (RR 1.56, 95% CI: 0.99 to 2.46, *p* = .06). In addition, only one study [13] reported that a lower incidence of fluid overload and metabolic acidosis was found in the group with early initiation of RRT.

**Table 4.** RRT-related complications.

	No. of studies	RR (95% CI)	*I* ^2^
Bleeding	6	0.89 (0.67, 1.17)	0
Catheter-related complications	4	1.56 (0.99, 2.46)	0
Fluid overload	1	0.11 (0.01, 0.89)	–
hypothermia	2	1.47 (0.35, 6.27)	0
Thrombocytopenia	2	1.12 (0.87, 1.44)	47%
Hyperkalemia	2	0.31 (0.02, 5.69)	77%
Metabolic acidosis	1	0.58 (0.35, 0.95)	–
Hypotension	4	1.07 (0.9, 1.28)	0
hypophosphatemia	2	2.34 (0.62, 8.82)	70%

### Sensitivity analysis

To assess the stability of the results of the current meta-analysis, we performed sensitivity analysis for mortality by removing the results of the most optimistic study [19], pessimistic study [9] or both, and statistically stable results were obtained, with a range of RR from 0.99 to 0.97. We also evaluated the effect on overall mortality of removing studies with unclear methodological quality [3,18,19,22,24,25,27] and found a similar result of mortality (RR 1.01, 95% CI: 0.91 to 1.12, *p* = .8) (*I*^2^ = 0).

### Publication bias

**Figure 6.**
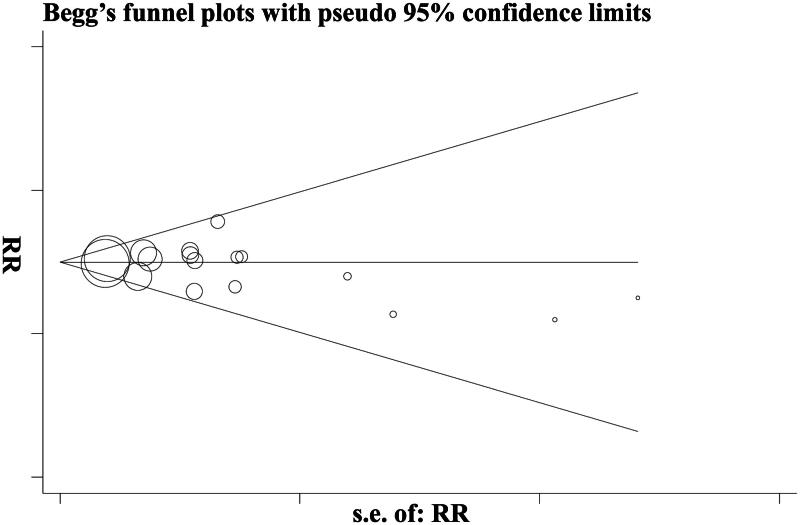
Illustration of publication bias.

No evidence of publication bias was detected for RR of mortality by either Begg’s funnel plots (*p* = .2) or Egger’s test (*t* − 0.93, *p* = .4) (Figure 6). There was also no publication bias for RR of need for RRT.

## Discussion

### Key findings

We performed a systematic review of the literature and identified 18 RCTs (more than 2800 patients) reporting the timing of initiation of RRT for critically ill patients with AKI. We found that without life-threatening complications, there was no difference in short-term (28-day) and long-term (>6-month) mortality between early and delayed initiation of RRT for patients with AKI. A similar mortality rate was found in critically ill AKI patients or community-acquired AKI patients, as well as in the subgroup of patients with sepsis or cardiac surgery. Furthermore, similar results were also found when CRRT or IHD was performed as the RRT modality. There was also no difference in the incidence of dialysis independence. A similar rate of complications was found for both approaches, with the exception that the early strategy might increase the incidence of catheter-related complications. Finally, and most importantly, more than one-third of patients in the delayed group did not receive RRT, but the early RRT strategy was associated with a significantly higher incidence of the need for RRT in AKI patients.

### Relationship to previous studies

As shown in Table 5, there were several previous meta-analyses [6,10,31–33] evaluating the timing of initiation of RRT for AKI patients, but their conclusions were controversial. Two meta-analyses [6,33], published in 2008 and 2011, both reported that an early RRT strategy could significantly improve the survival rate of AKI patients; however, more than 80% of the included studies in these two reviews were observational studies, which might be associated with potential allocation or selection bias. Wang’s review [31] also showed that ‘Early’ CRRT and IHD both could reduce the mortality compared with ‘late’ strategies, but the main problem was that 12 of 15 included studies were non-RCTs. A recent meta-analysis [10] included 7 RCTs and 27 observational studies that showed that although a significant survival benefit was found in low-quality studies (observational studies), no difference in survival rate was found in high-quality studies (mainly RCTs). Another review, published by Lai in 2017 [32], included only 9 RCTs and found a similar survival rate between an early strategy and a delayed strategy. However, the above two reviews failed to report the latest important RCTs [13,20,29,30] or RCTs published in Chinese [21,24,25,27]. In addition, all the previous meta-analyses did not present any information about different populations, such as critically ill or community-acquired AKI patients, for the RRT timing issue; furthermore, those meta-analyses also failed to present sufficient data on long-term mortality, dialysis independence, need for RRT or RRT-related complications.

**Table 5.** Comparison of previous meta-analysis.

	Our study	Lai [29]	Wierstra [8]	Wang [31]	Karvellas [4]	Seabra [30]
Year of publication		2017	2016	2012	2011	2008
Years of searching	1966–2018	–2016	1985–2015	1990–2011	1985–2010	1960–2006
Studies included	18	9	36	15	15	23
*RCTs*	18	9	7	3	2	5
Observational	0	0	29	12	13	18
Survival benefit	Negative	Negative	Negative	Favours early strategy	Favours early strategy	Favours early strategy

In contrast, the present systematic review includes data from 18 RCTs without language restriction, reporting the effect of timing of RRT for patients with AKI. Such a comprehensive meta-analysis appears more likely to accurately represent the timing of RRT for AKI patients and enables multiple subgroup analyses. First, in our review, we found a similar mortality rate in different populations and different modalities of RRT. Second, although there was no difference in mortality, we found that an early strategy was associated with a significantly higher incidence of the need for RRT, indicating that overtreatment of RRT might occur in clinical practice. Third, we found that an early RRT strategy might increase the incidence of catheter-related complications, which indicated that we should balance the advantages and disadvantages of RRT before the start of RRT.

As shown in Table 1, the populations in all included RCTs were adults. Unfortunately, there was no RCT focusing on the timing of RRT for pediatric AKI patients. Several recent observational studies [5,34,35] reported that early intervention with RRT could decrease the mortality rate in pediatric AKI patients. However, due to potential allocation or selection bias in observational studies, further high-quality, large RCTs for pediatric AKI patients are necessary, and future studies should focus on the identification of pediatric AKI patients who may benefit from early initiation of RRT.

### Identification of the timing of RRT

In the RCTs included in our review, there was tremendous variation in the criteria for the initiation of RRT in patients with AKI, such as AKI stage, duration from ICU admission or biochemical level. A limitation of this approach is that ‘early’ according to one study investigator might be considered ‘delayed’ by another study investigator. For instance, the delayed RRT protocol in the ELAIN study [11] was that RRT was performed within 12 h of diagnosis of KDIGO-AKI stage 3, which was similar to the early protocol in the AKIKI study (within 6 h of diagnosis of KDIGO-AKI stage 3) [12]. In addition, it is generally known that RIFLE, AKIN and KDIGO are approved as criteria for AKI stage, and in practice, clinicians often use them to evaluate the timing of RRT in AKI patients. The KDIGO-AKI guidelines have suggested that RRT should be considered at AKI stage 2; however, several cohort studies [36,37] have shown that very few patients with AKI stage 2 and only a minority of patients with AKI stage 3 receive RRT in clinical practice. In our review, 98% of AKI patients in the early group received RRT, and 65% in the delayed group received RRT, indicating that the relatively conservative RRT strategy was more closely aligned with clinical practice.

Thus, further RRT timing studies should use AKI staging rather than other investigator-defined criteria as the timing criteria to explore the best ‘cut-off’ timing of RRT for AKI patients. In our review of the ongoing RCTs on this topic registered with the NIH (www.clinical Trials.gov), one RCT (NCT01557361)[38] that uses RIFLE AKI staging to define the timing of early RRT may add to the knowledge base in this area.

### Strengths and limitations

This study comprehensively evaluated the effect of timing of RRT for AKI patients. Our search strategy was broad and included studies in English as well as Chinese. It included data from more than 2800 patients, 18 RCTs, and 9 countries from different regions of Asia, Europe and North America. Two independent investigators also rigorously assessed its methodological quality. Finally, we reported on multiple relevant outcomes.

However, our study also has several limitations. First, as described in Table 2, the features of timing of RRT in the two arms adopted in the included RCTs were varied. We accounted for potential heterogeneity by using a random-effects model; however, such modeling could not adjust for the possibility of ascertainment bias that is potentially associated with the use of variable investigator-defined RRT timing. Second, because of the nature of the intervention and logistical problems, the studies were not double-blinded. Although this might not influence the primary outcome (mortality), there is still potential for bias. Third, despite the inclusion of 18 RCTs, it must be noted that eight studies (44%) were small (fewer than 100 patients) and that not all included RCTs reported all relevant outcomes. For instance, 61% of the included RCTs reported the need for RRT, 50% reported the incidence of dialysis independence and 50% reported RRT-related complications. Thus, there might be outcome reporting bias in our study. Last, but not least, only published studies with selective databases were included for data analysis, and the unavailability of unreported outcomes could also have resulted in reporting bias. Regardless of these limitations, we sought to minimize bias throughout our study by strict method identification, data selection, and statistical analysis, as well as by controlling for publication bias and performing sensitivity analyses.

In conclusion, as no life-threatening complications occurred, the available RCTs did not show any benefit of early RRT strategies for critically ill or community-acquired AKI patients or in populations with sepsis or cardiac surgery recipients. In contrast, a delayed strategy might avert the need for RRT. Due to the limitation of the investigator-defined RRT timing, further high-quality RCTs using AKI staging for the timing criteria are desirable.

## Supplementary Material

Supplemental Material

Supplemental Material

Supplemental Material

## Geolocation information

No. 37 Guoxue Street, Chengdu, Sichuan, China.
