# Supplementary material for: Timing of initiation of renal replacement therapy in acute kidney injury: an updated meta-analysis of randomized controlled trials
Source: Ren Fail. 2020 Jan 2;42(1):77–88. doi: 10.1080/0886022X.2019.1705337 (PMC6968507; doi:10.1080/0886022X.2019.1705337)
Supplement: Supplemental Material [file IRNF_A_1705337_SM7411.docx]

Search strategy terms

| MEDLINE, searched 31/12/2018 | |
| --- | --- |
| 1 | Acute kidney injury [MeSH majr] OR “acute kidney”[ti] OR “acute renal”[ti] |
| 2 | Renal replacement therapy [MeSH majr] OR dialysis [ti] OR dialyzed [ti] OR dialyzing [ti] OR hemodialysis [ti] OR hemofiltration [ti] |
| 3 | Time to treatment [MeSH] OR Time factors [MeSH] OR Early [ti/ab] OR earlier [ti/ab] OR time [ti/ab] OR timing [ti/ab] OR accelerate [ti/ab] OR accelerated [ti/ab] OR accelerating [ti/ab] OR acceleration [ti/ab] OR late [ti/ab] |
| 4 | Random OR randomly OR randomized OR randomization |
| 5 | Combine #1 AND #2 AND #3 AND #4 |

| Embase, searched 31/12/2018 | |
| --- | --- |
| 1 | Acute kidney injury [MeSH majr] OR “acute kidney”[ti] OR “acute renal”[ti] |
| 2 | Renal replacement therapy [MeSH majr] OR dialysis [ti] OR dialyzed [ti] OR dialyzing [ti] OR hemodialysis [ti] OR hemofiltration [ti] |
| 3 | Time to treatment [MeSH] OR Time factors [MeSH] OR Early [ti/ab] OR earlier [ti/ab] OR time [ti/ab] OR timing [ti/ab] OR accelerate [ti/ab] OR accelerated [ti/ab] OR accelerating [ti/ab] OR acceleration [ti/ab] OR late [ti/ab] |
| 4 | Random OR randomly OR randomized OR randomization |
| 5 | Combine #1 AND #2 AND #3 AND #4 |

| the Cochrane Library, searched 31/12/2018 | |
| --- | --- |
| 1 | Acute kidney injury [ti/ab/kw] OR “acute kidney”[ ti/ab/kw ] OR “acute renal”[ ti/ab/kw ] |
| 2 | Renal replacement therapy [ti/ab/kw] OR dialysis [ ti or ab or kw ] OR dialyzed [ ti or ab or kw ] OR dialyzing [ ti or ab or kw ] OR hemodialysis [ ti or ab or kw ] OR hemofiltration [ ti or ab or kw ] |
| 3 | Time to treatment [ ti/ab ] OR Time factors [ ti/ab ] OR Early [ti/ab] OR earlier [ti/ab] OR time [ti/ab] OR timing [ti/ab] OR accelerate [ti/ab] OR accelerated [ti/ab] OR accelerating [ti/ab] OR acceleration [ti/ab] OR late [ti/ab] |
| 4 | Random [ti/ab/kw] OR randomly [ti/ab/kw] OR randomized [ti/ab/kw] OR randomization [ti/ab/kw] |
| 5 | Combine #1 AND #2 AND #3 AND #4 |

| Google Scholar , searched 31/12/2018 | |
| --- | --- |
| 1 | Acute kidney injury [ti] OR acute renal failure [ti] |
| 2 | Renal replacement therapy OR dialysis OR hemodialysis OR hemofiltration |
| 3 | Random OR randomly OR randomized OR randomization |
| 4 | Combine #1 AND #2 AND #3 AND #4 |

| CNKI , searched 31/12/2018 | |
| --- | --- |
| 1 | “急性肾损伤，篇名”or “急性肾衰竭，篇名” |
| 2 | “肾脏替代治疗，篇名或摘要” or “透析，篇名或摘要” or “血液净化，篇名或摘要” |
| 3 | “随机，摘要” or “随机对照，摘要” or “随机化，摘要” |
| 4 | Combine #1 AND #2 AND #3 AND #4 |
