# Supplementary material for: Timing of initiation of renal replacement therapy in acute kidney injury: an updated meta-analysis of randomized controlled trials
Source: Ren Fail. 2020 Jan 2;42(1):77–88. doi: 10.1080/0886022X.2019.1705337 (PMC6968507; doi:10.1080/0886022X.2019.1705337)
Supplement: Supplemental Material [file IRNF_A_1705337_SM7410.docx]

RRT modalities, fluid balance and SOFA score at baseline

| Studies | RRT modalities | Fluid balance | SOFA score |
| --- | --- | --- | --- |
| Barbar 2018 | CRRT (CVVHDF 40%, CVVH 40%, CVVHD 20%, or IHD CRRT 56% at start, CRRT alone 46%; IHD alone 34%, Combo CRRT and IHD 20% | 3L positive | 12.2 |
| Bouman 2002 | CRRT CRRT 100% at start, CRRT alone 100% | Positive | 10.3 |
| Combes 2015 | CRRT Early group: HVHF for 48h and then CVVHDF or IHD Late group: CVVHDF or IHD | NR | 12 |
| Durmaz 2003 | IHD | NR | NR |
| Gaudry 2016 | CRRT or IHD CRRT 44% at start, CRRT alone 32%; IHD alone 47%, Combo CRRT and IHD 21% | NR | 10.8 |
| Jamale 2013 | IHD | NR | 7.5 |
| Lu 2012 | CRRT CVVH 100% at start | NR | 13 |
| Lumletgul 2018 | CRRT CVVH 100% at start | 5L positive | 12 |
| Meersch 2017 | CRRT CVVHDF 100% at start, CRRT alone 71.4%; Change to SLEDD alone 22.3%; Change to IHD alone 1.8%; Combo SLEDD/IHD 4.5%. | NR | NR |
| Payen 2009 | CRRT CVVH 100% at start | 4L positive | NR |
| Pursnani 1997 | IHD | NR | <8 |
| Srisawat 2017 | CRRT CVVH 100% at start | NR | 9.28 |
| Sugahara 2004 | CRRT CVVHD 100% at start | NR | NR |
| Tang 2016 | CRRT CVVHDF 100% at start | NR | NR |
| Wald 2015 | RRT | 5L positive | 13 |
| Xiao 2016 | IHD | NR | NR |
| Yin 2018 | CRRT CVVH 100% at start | NR | NR |
| Zarbock 2016 | CRRT CVVHDF 100% at start, CRRT alone 71.4%; Change to SLEDD alone 22.3%; Change to IHD alone 1.8%; Combo SLEDD/IHD 4.5%. | 6L positive | 16 |

NR, not reported
